# *Klebsiella pneumonia* in Sudan: Multidrug Resistance, Polyclonal Dissemination, and Virulence

**DOI:** 10.3390/antibiotics12020233

**Published:** 2023-01-21

**Authors:** Einas A. Osman, Maho Yokoyama, Hisham N. Altayb, Daire Cantillon, Julia Wille, Harald Seifert, Paul G. Higgins, Leena Al-Hassan

**Affiliations:** 1 Bioscience Research Institute, Ibn Sina University, Khartoum 11111, Sudan; 2 Department of Global Health and Infection, Brighton & Sussex Medical School, Brighton BN1 9PX, UK; 3 Department of Biochemistry, Faculty of Sciences, King Abdulaziz University, Jeddah 21589, Saudi Arabia; 4 Department of Tropical Disease Biology, Liverpool School of Tropical Medicine, Liverpool L3 5QA, UK; 5 Institute for Medical Microbiology, Immunology and Hygiene, Faculty of Medicine and University Hospital Cologne, University of Cologne, 50935 Cologne, Germany; 6 German Center for Infection Research (DZIF), Partner Site Bonn-Cologne, 50935 Cologne, Germany; 7 Center for Molecular Medicine Cologne, Faculty of Medicine and University Hospital Cologne, University of Cologne, 50931 Cologne, Germany

**Keywords:** *Klebsiella pneumoniae*, multidrug resistance, transmission, Sudan

## Abstract

The emergence and global expansion of hyper-virulent and multidrug resistant (MDR) *Klebsiella pneumoniae* is an increasing healthcare threat worldwide. The epidemiology of MDR *K. pneumoniae* is under-characterized in many parts of the world, particularly Africa. In this study, *K. pneumoniae* isolates from hospitals in Khartoum, Sudan, have been whole-genome sequenced to investigate their molecular epidemiology, virulence, and resistome profiles. Eighty-six *K. pneumoniae* were recovered from patients in five hospitals in Khartoum between 2016 and 2020. Antimicrobial susceptibility was performed by disk-diffusion and broth microdilution. All isolates underwent whole genome sequencing using Illumina MiSeq; cgMLST was determined using Ridom SeqSphere+, and 7-loci MLST virulence genes and resistomes were identified. MDR was observed at 80%, with 35 isolates (41%) confirmed carbapenem-resistant. Thirty-seven sequence types were identified, and 14 transmission clusters (TC). Five of these TCs involved more than one hospital. *Ybt9* was the most common virulence gene detected, in addition to some isolates harbouring *iuc* and *rmp1*. There is a diverse population of *K. pneumoniae* in Khartoum hospitals, harbouring multiple resistance genes, including genes coding for ESBLs, carbapenemases, and aminoglycoside-modifying enzymes, across multiple ST’s. The majority of isolates were singletons and transmissions were rare.

## 1. Introduction

*Klebsiella pneumoniae* is an important global pathogen causing a variety of infections in community and healthcare-associated infections, such as pneumonia, urinary tract infections (UTI), and bloodstream infections. It poses a serious threat to human health and is one of the six highly virulent and antibiotic resistant bacterial pathogens: *Enterococcus faecium*, *Staphylococcus aureus*, *Klebsiella pneumoniae*, *Acinetobacter baumannii*, *Pseudomonas aeruginosa*, and *Enterobacter* spp. (ESKAPE), requiring urgent global attention [1]. Multidrug resistance and carbapenem resistance in *K. pneumoniae* (MDR-Kp and CR-Kp) is of particular concern as treatment options are limited. 

The population structure of *K. pneumoniae* appears to be diverse yet highly structured, with distinct clonal groups (CG) subdivided into MDR- and hypervirulent (Hv)-clones [2]. A subset of these clones contribute to global diseases and outbreaks and are referred to as ‘global problem clones’, involving the transfer and spread of antibiotic resistance genes and endemic plasmids in highly disseminating successful clones worldwide [3]. 

Antimicrobial resistance (AMR) is a global health issue, with low- and middle-income countries (LMICs) carrying the largest burden of infection and AMR, both in the community and healthcare settings [4,5]. Global efforts such as the World Health Assembly’s Global Action Plan on AMR (GAP-AMR), which was followed by the adoption of National Action Plans (NAP)s by member countries, and the World Health Organisation (WHO) Global Antimicrobial Resistance and Use Surveillance System (GLASS) are all strategies to contain AMR [6]. However, lack of systematic surveillance and scarcity of data on the burden of infectious diseases and AMR is largely due to limited infrastructure and financial support. 

Sudan is a lower middle-income country in Africa, with a population of approximately 44 million (in 2021) covering an area of 1.8 million km^2^. Median age is 19.7, and life expectancy at birth is 64.1 years [7]. Sudan’s health care system is fragile, hospital-centric, and fragmented in both the public and private sectors, and facing several challenges. The WHO data show that health-associated out-of-pocket expenditure paid by households are ~74%, which cover curative care, medicines, and medical consumables [7]. There is disparity in the distribution of health care personnel between the public and private sectors and between urban and rural areas. Moreover, the high turnover and migration of health professionals continue to threaten the capacity to respond to the increased demand for health services [7]. An analysis of the AMR situation by the Sudanese NAP revealed high rates of resistance, and an urgent need to address the situation by improving awareness, surveillance, hygiene, and infection control, and the optimization of antimicrobial medicines (https://www.who.int/publications/m/item/sudan-national-action-plan-on-antimicrobial-resistance; last accessed on 7 January 2023). However, the implementation of antimicrobial stewardship and improving the access to antimicrobials is difficult due to the lack of supervisory systems to ensure rational prescribing, combined with poor accessibility of essential antibiotics in rural areas. 

MDR *K. pneumoniae* is on the WHO global priority pathogen list [8], but despite global efforts there are significant gaps in robust epidemiological data, particularly in LMICs. Recognising the significance of MDR- and CR-Kp globally and the need for accurate epidemiological typing of the organism, it is important to gain insight into circulating clones in order to contextualise data on national and international lineages, and to inform locally-relevant infection prevention and control based on local data. In Sudan, *K. pneumoniae* is highly prevalent in hospitals and has been reported in numerous studies as the most common Gram-negative organism identified [9,10,11]. *K. pneumoniae* was the second most frequent organism contributing to urinary tract infection (UTI) in diabetic patients [12]. In a study on the presence of pathogenic bacteria isolated from bank notes in Khartoum, *K. pneumoniae* was the most prevalent organism identified [13]. Despite this prevalence, few studies have included molecular methods for isolate identification, confirmation, or resistance surveillance. We have previously conducted a study on the local epidemiology of CR-Kp in Khartoum by using MLST [14]. In this study we used whole genome sequencing (WGS) to generate more robust data on the molecular epidemiology, transmission, resistome, and virulence profiles of MDR *K. pneumoniae* from multiple hospitals in Khartoum, Sudan. 

## 2. Results

**Figure 1:**
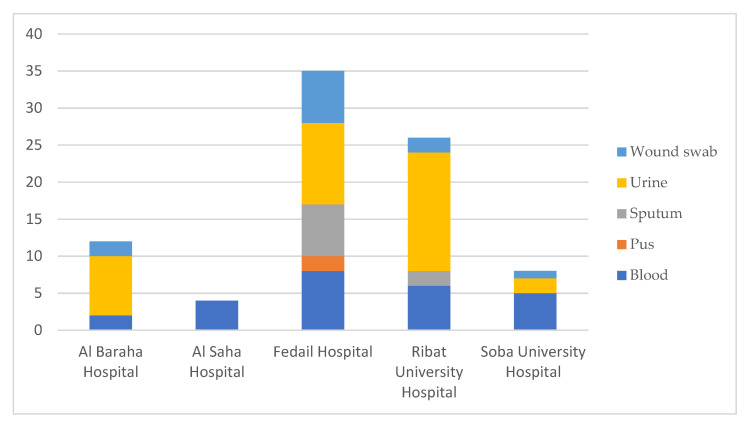
Details of isolate sources and hospitals.

A total of 86 isolates were included in this study from five different hospitals in the Sudanese capital of Khartoum: 2 isolates from 2016, 24 from 2018–2019, and 60 from March–September 2020 (detailed sampling procedure in the Methods). Eighty-four *K. pneumoniae* isolates were confirmed by MALDI-TOF and Kleborate, while 2 isolates (LH_F129 and LH_F72) were identified as *Klebsiella quasipneumoniae* subsp. *quasipneumoniae* and *Klebsiella variicola* subsp. *variicola*, respectively. The organisms came from a variety of samples: blood (*n* = 25, 29.4%), pus (*n* = 2, 2.4%), sputum (*n* = 9, 10.6%), urine (*n* = 37, 43.5%), and wound swabs (*n* = 12, 14.1%) (Figure 1; Table 1).

Most isolates came from Fedail Hospital, followed by Ribat University Hospital, both of which are two of the largest hospitals in Khartoum. Urine and blood samples were most common across all isolates.

Sixty-eight isolates (80%) were MDR (resistant to >3 antibiotic classes), 35 of which (51%) were carbapenem resistant (imipenem andor meropenem MIC ≥ 4 mgL). The remaining 16 isolates were susceptible to all tested antibiotics. All isolates were colistin susceptible (MIC < 2 mgL). Multiple resistance mechanisms were identified (Table 2 and Supplementary Table S1), contributing to the observed MDR phenotypes. Aminoglycoside resistance was present in 49 isolates (57%), mediated by multiple aminoglycoside-modifying enzymes. β-lactam resistance was detected in 58 isolates and was mediated by acquired β-lactamases: OXA-1 (*n* = 19), OXA-9 (*n* = 4, two of which were co-harbouring OXA-1 and TEM-1), CMY (*n* = 3, one of which was co-harbouring OXA-1), DHA-1 (*n* = 3, two of which were co-harbouring TEM-1), and SCO-1 (*n* = 3, one of which was co-harbouring TEM-1); TEM-1 was the most prevalent β-lactamase, identified in 40 isolates. The acquired extended-spectrum β-lactamase (ESBL) CTX-M-15 was present in 64 isolates, and CTX-M-14 was present in four isolates, three of which co-harboured CTX-M-15. Seventeen isolates did not harbour any ESBLs. SHV-variants were present in all *K. pneumoniae* isolates. 

Carbapenem resistance was mediated by NDM-1 (*n* = 23), NDM-4 (*n* = 1), and NDM-5 (*n* = 11, three of which co-harboured OXA-48); one isolate harboured OXA-48 on its own, and one isolate harboured OXA-232 (OXA-48-like enzyme). Three isolates displayed phenotypic carbapenem resistance, but no acquired carbapenemases were detected, and the observed carbapenem resistance was associated with a combination of an ESBL and modifications in the OmpK35OmpK36 (Table 2 and Table S1). LH_F164 possessed a truncated *bla*_NDM-1_ gene but also harboured OXA-1, CTX-M-15, and a nucleotide substitution (G690A) in the OmpK35 encoding gene, leading to a premature stop codon. LH_R313, on the other hand, lacked an *ompK35* gene, and carbapenem resistance was mediated by a modification of OmpK36 containing an LSP insertion in the amino acid sequence at position 184.

**Figure 2:**
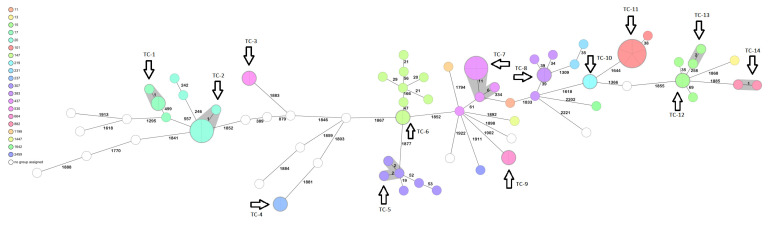
Ridom SeqSphere+ minimum spanning tree (MST) for 84 samples based on 2358 columns, pairwise ignoring missing values, logarithmic scale, *K. pneumoniae* MLST Pasteur (7). Cluster distance threshold: 15. Isolates are grouped by colour, indicating the different STs. Thirty-seven different STs were identified, in addition to 14 transmission clusters, represented by shaded nodes and arrows. Numbers between the nodes indicate the number of allelic differences.

A total of 37 different sequence types (STs) were identified in the study, highlighting the epidemiological diversity of the isolates. We observed 14 small transmission clusters (TC) by cgMLST, with isolates differing by 0–5 alleles (Figure 2). TC-1 (ST17) comprises three isolates collected from Al-Saha Hospital (ASH) in 2019; however, they differed in their respective resistomes. Metadata relating to the location of patients or date of isolation are not available for these isolates. LH_HishK11 and LH_HishK13 are both carbapenem- susceptible, while LH_HishK12 is MDR and harbours multiple aminoglycoside-modifying enzymes (*aac(3)-IIa-like;aac(6’)-Ib-cr-like;aadA5*) and β-lactamases: *bla*_CTX-M-15_, *bla*_OXA-1_, and *bla*_NDM-1_. All other TCs comprise isolates with identical resistomes (Figure 2 and Table S1).

The most frequently isolated ST in the study was ST147 (*n* = 9), followed by ST20 (*n* = 7), ST437 (*n* = 7), ST101 (*n* = 6), ST15 (*n* = 6), ST307 (*n* = 6), ST383 (*n* = 5), and ST17 (*n* = 4), all of which are high-risk global clones (GC), defined as epidemic high-risk clones over-represented globally [2], with the remaining 29 singleton STs. Isolates within the same ST exhibited some similarities at the genomic level, but as seen in Table 2 and Table S1, some had different resistance phenotypes and genes. For example, ST147 isolates came from multiple hospitals, with isolates from FH all collected within a 1-month timeframe (July–August 2020), but they differed by ≥21 alleles and transmission was ruled out. Moreover, differences were seen in their virulence and resistome profiles, with LH_F134 harbouring no recognised virulence factors, while LH_F149, LH_F18, and LH_F97 all harboured *ybt9* on a ICEKp3 structure. LH_F134, LH_F149, and LH_F18 were carbapenem resistant, mediated by *bla*_NDM-1_; however, LH_F97 was carbapenem susceptible. Similar data are observed in ST-147 isolates from RUH which had identical resistomes, except R344, which harboured *bla*_NDM-5_ and not *bla*_NDM-1_ and differed by >50 allelic differences per the cgMLST and was therefore not part of a single transmission cluster. The only TC in this group were two identical isolates from Al Baraha Hospital, which also had identical resistomes.

ST20 was also found in multiple hospitals (SUH, ASH, ABH, and RUH), and comprised one TC from two hospitals (TC-2; Figure S1), but there were differences in their resistome. All ST437 were MDR and contained the hypervirulence gene *ybt9* on an ICEKp3 structure. This ST comprised four identical isolates forming a TC (TC-2) with two other isolates, and a singleton. Key resistome differences were observed in aminoglycoside-modifying enzymes and carbapenemases: LH_K4 (collected in 2018 from ABH) had *aac(3)-IId-like;aac(6’)-Ib’.v1;aadA;aph(3’)-VI* and *bla*_NDM-1_, whereas the remaining isolates (collected from FH in 2020) all had *rmtB* and *bla*_NDM-5_. The imipenem MIC was also notably less for LH_K4, harbouring *bla*_NDM-1_ at 64 mgL vs. >128 mgL for the remaining NDM-5 isolates.

ST101 was only present in isolates in 20182019 (SUH, BH; Supplementary Figure S1) and comprised five identical isolates (TC-11) and a singleton. All ST-101 isolates were MDR, but only two were carbapenem-resistant: LH_HishK5 and LH_HishK7, mediated by NDM-1 (LH_HishK5) and modification in *ompK35* (G947A, leading to a premature stop codon at position 316), combined with *bla*_OXA-1_ and *bla*_CTX-M-15_ (LH_HishK7). 

All ST15 isolates were MDR (and comprised two, TC-12 and TC-13, and two singletons), but were carbapenem-susceptible, except for LH_F164, which was carbapenem-resistant (imipenem MIC 16 mgL), associated with a G690A substitution in the *ompK35* gene (leading to a premature stop codon) combined with *bla*_OXA-1_. LH_F164 was the only strain which was also positive for *ybt9* (on ICEK3p) within this ST. Isolates within ST307 came from two different hospitals (RUH and FH), with 56 isolates from RUH. Three of these isolates form a transmission cluster. Interestingly, one isolate, collected 4 months later in July 2020 (LH_R275) from RUH appears to have lost the MDR and CR pheno- and geno-types.

The ST-383 isolates were all MDR, CR, and were comprised of three singletons from Fedail Hospital, and one TC from Soba University Hospital (TC-8). Of these, 35 (LH_HishK8, LH_HishK9, and LH_F190) isolates co-harboured multiple resistance genes: *bla*_CTX-M-14_ and *bla*_CTX-M-15_, and *bla*_NDM-5_ and *bla*_OXA-48_. The remaining two isolates (LH-F15 and LH-F35) harboured either *bla*_CTX-M-14_ and *bla*_OXA-48_ or *bla*_CTX-M-15_ and *bla*_NDM-5_, respectively. Of the three ST17 isolates, only one was MDRCR and harboured *bla*_NDM-1_. 

Of the remaining 29 singleton STs, 11 of them were MDR and CR, whereas the remaining 18 were susceptible to all tested antibiotics. 

Thirty-six isolates harboured one or more virulence genes, namely *ybt9*, *ybt10*, *ybt4*, *ybt14*, *iuc*, *rmp1*, *KpVP-1*, and *rmpA2* (Table 2 and Table S2). Yersiniabactin *ybt9* located on ICEKp3 was the most common structure detected (*n* = 21), found in ST147, ST101, ST15, and ST437 (all of which are GCs), as well as ST11, and was present in multiple isolates and hospitals in our collection. Seven isolates harboured *ybt10* on ICEKp4, associated with ST530, ST307 (GC), ST3161, and ST45. Aerobactin *iuc1* was associated with ST383 (*n* = 4, of which two co-harboured *rmp1*), and 1 ST231 co-harboured *ybt14* and *iuc*. 

Forty different K-loci were identified, as detailed in Supplementary Table S2, along with seven variants of O-loci, most common of which were O1 (*n* = 30) and O2 (*n* = 21). The O-locus variant could not be identified in 10 isolates. 

## 3. Discussion

The aim of this study was to conduct a snapshot analysis of the molecular epidemiology of the *K. pneumoniae* population from hospitals in Khartoum, Sudan. We have included isolates from five hospitals through several sampling timeframes over the years. *K. pneumoniae* was implicated in multiple infections, including blood, skin and soft tissue (SST), and urine. The majority of isolates (81%) were MDR, of which more than half were also carbapenem resistant, mediated mainly by NDM-1-, NDM-5-, and OXA-48-like enzymes. Interestingly, KPC was absent from the isolate collection, despite its global prevalence in CR-Kp, particularly in ST258. Carbapenem resistance was also mediated by the presence of an ESBL and modifications in OmpK35 and OmpK36 in three isolates (Table 2). Modifications in outer-membrane porins which restricts antibiotic entry is an important carbapenem resistance mechanism [15]. *OmpK35* is usually truncated due to a mutation encoding a frame shift that results in a premature stop codon [16,17]. *OmpK36* is more heterogenous, rarely truncated, and resistance mutations are common leading either to abundance of OmpK36 in the outer membrane or constriction of the pore size [15,18]. Carbapenem MIC for LH_R290 showed intermediate resistance (IMI 4 mgL) without an acquired carbapenamase; however, the isolate harbours SCO-1, a plasmid-encoded ESBL able to hydrolyse not only penicillins but also, to a lesser degree, cephalosporins and carbapenems. Since its discovery in 2007, the *bla*_SCO-1_ gene (GenBank accession no. EF063111) has been identified in *Acinetobacter baumannii*, *Escherichia coli*, *Serratia marcescens*, *Klebsiella aerogenes*, *Salmonella enterica*, and only four *K. pneumoniae* isolates [19]. 

The epidemiological analysis of *K. pneumoniae* in Sudan revealed a large diversity of 37 different STs, with 13 transmission clusters (Figure 1). Five of these clusters have been collected from different hospitals, thereby indicating intra-hospital circulation of clones. Global problem high-risk MDR clones (GC), identified by Wyres et al., 2020, have been found in this study; however, we noted that not all were MDR. We have identified multiple losses of resistance within these GCs over time. For example, isolates in ST307 at RUH collected in FebruaryMarch 2020 were carbapenem-resistant; however, isolate LH_R275 collected in July 2020 was neither MDR nor CR. Similarly, isolates within ST147: LH_F134, LH_F149, LH_F18, were CR harbouring *bla*_NDM-1_; however, LH_F97 (isolated from the same hospital) is carbapenem-susceptible, but had acquired the *ybt9* virulence gene on ICEKp3. ST437 isolates appear to have lost *bla*_NDM-1_ (from 2018) and acquired *bla*_NDM-5_ in the isolates collected in 2020. However, we did not specifically look for plasmids in this study, and further detailed investigations of possible plasmid acquisition and loss events must be conducted to confirm the observed results. The differences in the resistomevirulomes of isolates within the same ST and transmission cluster is important to note for outbreak and epidemiological studies. The data provided by genomic analysis are an important tool where molecular epidemiology is combined with patient clinical data and the resistomes to obtain accurate information on transmission of resistant and virulent pathogens. 

Yersinibectin (*ybt9* and *ybt10*) were the most identified virulence genes in the study and were mostly associated with GCs: ST101, ST147, and ST307, in addition to their occurrence in singleton non-GCs, such as ST39, ST437, and ST882 (Table 2). *ybt* is usually present in 30–40% of *K. pneumoniae* human HAI isolates and up to 13% of community-acquired isolates [20]. Other virulence genes also identified in the study included aerobactin, *iuc1*, and hypermucoidy-associated *rmpA* genes. We have noted AMR-hypervirulence convergence events in 24 isolates, which harboured a virulence gene and were MDR: ST530, ST11, ST147, ST101, ST231, ST307, ST15, ST383, ST437, and ST45. It is important to note that we have not confirmed the virulence phenotypes of the isolates, and more research into the clinical significance of hypervirulence is required. 

ST383 isolates were particularly unique in their resistomes and virulomes. The isolates co-harboured multiple ESBLs and carbapenemases simultaneously, CTX-M-14 and CTX-M-15, in addition to NDM-5 and OXA-48 in the same isolates and *iuc-1*, *rmp1* on KpVP-1, and *rmpA2*. ST383 is a prevalent MDR clone in China, Australia, the UK, and Germany [21]. In a study from Egypt, ST383 was identified in a single isolate simultaneously encoding CTX-M-14 (on an IncLM plasmid) and CTX-M-15 (encoded on a hybrid IncHI1BIncFIB plasmid) in addition to both NDM-5 and OXA-48 [22]. Results by PlasmidFinder show the same plasmids are present in our ST383 isolates (incFIBIncHI1B and incL).This clearly indicates the endemicity of this particular clone in the region, as the data presented in the Egyptian isolate is identical to that in our ST383 cluster. 

In our previous study on *K. pneumonia* isolated in Khartoum, 2015–2016, we performed MLST on 117 isolates. All were MDR, and 42.8% were CR-Kp [14]. A similar diversity was observed in the current study, with 52 different STs, with the most common being ST383 (*n* = 8), ST101 (*n* = 5), and ST48 (*n* = 2), in addition to assignment of 15 novel STs. NDM was the most common carbapenemase, in addition to VIM, which has not been detected in the current study. A study conducted in Sudan by Adam et al. from 2015–2016 [23] revealed that metallo-β-lactamases (VIM and IMP) were prevalent in *K. pneumoniae* (1520 isolates), and found in combination with NDM in 520 isolates, in contrast to our study where VIM and IMP were not detected and NDM was the most prevalent MBL. Comparing the data from these studies indicates the maintenance of ST101, ST383, ST219, and ST437 lineages in Khartoum hospitals over the years, and the presence of novel lineages, as reported in this study. 

In comparison to the present study, we also investigated *A. baumannii* from several different hospitals in Khartoum during the same time period and found that the *A. baumannii* isolates were mostly transmissions and very few were singletons [24]. This highlights that even in an area that obviously has a high incidence of hospital-acquired infections and patient-to-patient transmissions, including between hospitals, *K. pneumoniae* is not easily transmitted. While most *K. pneumoniae* in this study were not transmissions, they do harbour similar carbapenemases, which may indicate transmission of resistance genesmobile genetic elements, which will be the focus of a follow-up study. 

When compared to other African studies, the *K. pneumoniae* population in Southern Nigeria was found to contain four dominant lineages: ST307, ST524, ST15, and ST25, while CR remained low at 8%, with no isolate carrying a combination of carbapenemases, in contrast to our study, where carbapenem-resistance was much higher (41.6% of 84 isolates) and three strains co-harboured NDM and OXA-48 [25]. A multi-centre pilot study in Egypt revealed that ST11, ST147, ST231, ST383, and ST101 were prevalent, which is similar to our study [26]. The observed similarities and difference with the local, regional, and international studies highlights the diversity and dynamic nature of the representative local population of *K. pneumoniae* as well as the potential for regional dissemination of clones in different countries, and the need to support local capacity in robust epidemiological surveillance. The first WGS study on a single ST14 *K. pneumoniae* from Sudan was conducted in 2019 [27]. To the best of our knowledge, this is the first WGS-based study of a large collection of *K. pneumoniae* from Sudan. 

Through this study, we aimed to generate robust epidemiological data on *K. pneumoniae*; albeit acknowledging some limitations. We have tried to collect as many *K. pneumoniae* isolates within the indicated timeframes. However, we cannot exclude sampling bias (such as missed samples or misidentification), as the study team was not directly involved in the sampling procedures. Routine sampling of infected patients is not always performed in local hospitals in Sudan, and sampling is frequently sporadic and based on individual doctors’ rather than hospital policiesguidelines due to poor microbiological infrastructure in hospitals. The fragmented health service, and the large out-of-pocket contribution by individuals and households, as presented in the introduction, lead to some hospitals also outsourcing microbiology to private laboratories. This study, however, focused on data from hospital-based laboratories only. Furthermore, the study was interrupted during COVID-19 lockdowns, so isolates between April and July 2020 were not collected. Some associated demographic data were also lacking for some isolates collected in 2018–2019. The sampling strategy therefore cannot exclude missed transmissions. Nevertheless, this study provides a starting point for the understanding of the population structure and diversity of *K. pneumoniae* in Khartoum, Sudan. It furthermore supports national and global efforts in providing robust epidemiological information on important HAI. This study highlights the importance of using WGS in AMR surveillance. Despite the vast possibilities for implementation, major challenges relating to capacity and training, and particularly analysis, of WGS still exist in many LMICs [28].

## 4. Materials and Methods

A total of 86 non-repetitive isolates were collected within these timeframes: Twenty-four isolates in 2018–2019 from Al-Baraha Hospital (ABH), Al-Saha Hospital (ASH), and Soba University Hospital (SUH); then 60 isolates in March–September 2020 from Fedail Hospital (FH), Ribat University Hospital (RUH), and SUH. Two additional isolates (from 2016) were included from RUH. Descriptive statistics was used to summarise the details of each isolate, including date of isolation, sample type, and hospital (Figure 1 and Table 1). The clinical microbiology laboratories in the named hospitals identify clinical specimens to the genus levels by conventional phenotypic and biochemical methods (growth and colony morphology on various media, and biochemically: indole negative, MR negative, VP positive, citrate positive, oxidase negative, and catalase positive). No specific selection criteria were implemented, as we aimed to collect and characterise any *K. pneumoniae* isolates identified in the hospitals during the study periods. Upon identification of *K. pneumoniae* by the clinical microbiology laboratories, the isolates were stored at 4 °C and collected by the study team within 48 h. It is important to note that the study team cannot guarantee any misidentification or loss of samples that were not submitted to the study.

All acquired isolates were subsequently confirmed phenotypically by conventional culture methods to exclude any contamination, then genotypically by amplification of 16S-23S rDNA internal transcribed spacer (ITS) of *K. pneumoniae*, as described in our preceding study [29]. Further identification confirmation was performed by MALDI-TOF prior to WGS. 

Antimicrobial susceptibility (AST) was initially performed by disk-diffusion at the clinical microbiology laboratories and interpreted according to CLSI guidelines [30]. Upon confirmation of species, as described above, the minimum inhibitory concentration (MIC) was determined by MICRONAUT-GN, (Merlin Diagnostika, Germany). This system allows the determination of the MIC for a panel of Gram-negative specific antibiotics: meropenem (MER), gentamicin (GEN), amikacin (AMK), trimethoprim-sulfamethoxazole (SXT), ciprofloxacin (CIP), colistin (COL), Amoxicillin (AMX), AmoxicillunClavulanate (AMC), Cefotaxime (CTX), Ceftazidime (CAZ), Cefuroxim (CXM), Etrapenem (ERT), and Temocillin (TMO), with two concentrations (mgL) based on the EUCAST MIC breakpoints for sensitivity (S) or resistance (R) in a single microtiter plate. Additionally, MIC for imipenem (IMI) was performed by broth microdilution according to the EUCAST guidelines V2, 2020 [31]. Quality control strains *E*. *coli* ATCC 13846, ATCC 12241, and *P. aeruginosa* ATCC12903 were used in all AST experiments. Multidrug resistance was defined as resistance to 3 or more antimicrobial classes. 

Total DNA was extracted from the bacterial isolates using a MagAttract HMW DNA Kit (Qiagen, Hilden, Germany), according to the manufacturer’s instructions. Sequencing libraries were prepared using the Nextera XT library prep kit (Illumina GmbH, Munich, Germany) for a 250-bp paired-end sequencing run on an Illumina MiSeq platform. De novo assembly was constructed using Velvet v1.1.04. Molecular epidemiology of the isolates was investigated by core-genome MLST (cgMLST) using Ridom^®^ SeqSphere^+^ version 8.5.1 [32]. 

Kleborate v2.0.4 was used to screen genome assemblies for Sequence Types (STs), capsular type, and virulence loci [33]. The resistome for the assembled genomes was identified using Kleborate v2.0.4 and the Resfinder database v3.2 https://cge.cbs.dtu.dkservicesResFinder (accessed on 10 February 2021) [34]. Sequence alignment and visualisation was performed by Geneious Prime. 

All assembled genome sequences were submitted to Genbank under BioPproject ID PRJNA912084. 

## 5. Conclusions

This study aims to provide a population snapshot of *K. pneumoniae* in Sudan. ST101, ST147, and ST437 are epidemic and predominantly present in multiple hospitals. MDR and CR-Kp were also prevalent (80% and 50%, respectively), which is alarming. The detection of multiple virulence genes and the potential emergence of AMR-hypervirulence convergence events must also be considered for local surveillance. We conclude that a diverse population of *K. pneumoniae* is present in hospitals in Khartoum, Sudan. Further genomic investigations, and inclusion of more data from national and regional hospitals, would enable delivery of value on a local, national, regional, and global level in understanding the pathogen dynamics.

## Figures and Tables

**Table 1:** Isolate details: date and type of sample, and hospital.

Sample Name	Collection Date	Hospital	Isolation Source	Additional Information
HishK1	2016	RUH	Urine	
HishK2	2016	RUH	Blood	NICU
HishK10	2018	SUH	Blood	NICU
HishK3	2018	SUH	Urine	
HishK4	2018	SUH	Urine	
HishK5	2018	SUH	Blood	NICU
HishK7	2018	SUH	Blood	NICU
HishK8	2018	SUH	Blood	NICU
HishK9	2018	SUH	Wound swab	
K10	2018	ABH	Wound swab	
HishK11	2019	ASH	Blood	
HishK12	2019	ASH	Blood	
HishK13	2019	ASH	Blood	
HishK14	2019	ASH	Blood	
K11	2019	ABH	Urine	
K12	2019	ABH	Urine	
K13	2019	ABH	Wound swab	
K15	2019	ABH	Urine	
K2	2019	ABH	Urine	
K3	2019	ABH	Blood	NICU
K4	2019	ABH	Urine	
K5	2019	ABH	Urine	
K6	2019	ABH	Blood	
K8	2019	ABH	Urine	
K9	2019	ABH	Urine	
LH_R146	08 February 2020	RUH	Urine	
LH_S25	23 February 2020	SUH	Blood	
LH_R100	26 February 2020	RUH	Sputum	ICU
LH_R92	28 February 2020	RUH	Urine	
LH_R107	01 March 2020	RUH	Urine	NICU
LH_R120	03 March 2020	RUH	Urine	
LH_R154	11 March 2020	RUH	Urine	Outpatient
LH_R167	18 March 2020	RUH	Blood	NICU
LH_R182	19 March 2020	RUH	Blood	Inpatient
LH_R195	22 March 2020	RUH	Blood	ICU
LH_R164	23 March 2020	RUH	Sputum	Inpatient
LH_R174	23 March 2020	RUH	Wound swab	Inpatient
LH_R162	23 March 2020	RUH	Urine	Outpatient
LH_R208	26 March 2020	RUH	Urine	Outpatient
LH_R223	28 March 2020	RUH	Wound swab	Outpatient
LH_R219	28 March 2020	RUH	Urine	Outpatient
LH_R275	12 July 2020	RUH	Urine	Inpatient
LH_R289	18 July 2020	RUH	Blood	Outpatient
LH_R290	27 July 2020	RUH	Urine	Urology unit
LH_R314	30 July 2020	RUH	Blood	
LH_R313	06 August 2020	RUH	Urine	
LH_R323	08 August 2020	RUH	Urine	
LH_R344	12 August 2020	RUH	Urine	
LH_F2	16 August 2020	FH	Urine	
LH_F15	16 August 2020	FH	Sputum	
LH_F18	17 August 2020	FH	Sputum	
LH_F25	17 August 2020	FH	Blood	
LH_F50_1	18 August 2020	FH	Urine	
LH_F35	18 August 2020	FH	Urine	
LH_F68	20 August 2020	FH	Wound swab	
LH_F64	20 August 2020	FH	Wound swab	
LH_F66	20 August 2020	FH	Urine	
LH_F82	21 August 2020	FH	Pus	
LH_F86	21 August 2020	FH	Urine	
LH_F97	22 August 2020	FH	Urine	
LH_F101	22 August 2020	FH	Urine	
LH_R384	22 August 2020	RUH	Urine	
LH_F104	23 August 2020	FH	Urine	
LH_F102	23 August 2020	FH	Blood	
LH_F122	25 August 2020	FH	Blood	
LH_F126	25 August 2020	FH	Wound swab	
LH_F139	26 August 2020	FH	Wound swab	
LH_F134	26 August 2020	FH	Blood	
LH_F137	27 August 2020	FH	Sputum	
LH_R387	27 August 2020	RUH	Urine	
LH_F143	27 August 2020	FH	Blood	
LH_F146	28 August 2020	FH	Urine	
LH_F149	29 August 2020	FH	Pus	
LH_F164	01 September 2020	FH	Wound swab	
LH_F158	01 September 2020	FH	Blood	
LH_F159	01 September 2020	FH	Blood	
LH_F169	02 September 2020	FH	Blood	
LH_F174	02 September 2020	FH	Wound swab	
LH_F175	03 September 2020	FH	Wound swab	
LH_F176	03 September 2020	FH	Sputum	
LH_F190	05 September 2020	FH	Sputum	
LH_F192	05 September 2020	FH	Sputum	
LH_F281	20 September 2020	FH	Urine	

RUH: Ribat University Hospital, SUH: Soba University Hospital, ASH: Al-Saha Hospital, FH: Fedail Hospital, ABH: Al-Baraha Hospital, NICU: neonatal intensive care unit.

**Table 2:** Resistome and Virulence details of STs.

ST	ESBL *bla*	Carb R *bla*	Intrinsic β-Lactamase *bla*	Virulence
101 (6)	*CTX-M-15* (6)	*NDM-1* (1)*; ompK* substitution (1)	*SHV-1*	*ybt9* (6)
11 (1)	*CTX-M-15*	*NDM-4*	*SHV-11*	*ybt9*
1198 (1)	*-*	*-*	*SHV-11*	
13 (1)	*CTX-M-15*	*-*	*SHV-1*	
1147 (1)	*-*	*-*	*SHV-27*	
147 (9)	*CTX-M-15* (9)	*NDM-1* (5); *NDM-5* (1)	*SHV-11*	*ybt9* (6)
ST15 (6)	*CTX-M-15* (5)	*OmpK35* substitution (1)	*SHV-28*	*ybt9* (1)
ST17 (4)	*CTX-M-15* (2)	*NDM-1*	*SHV-11*	
ST20 (7)	*CTX-M-15* (7)	*NDM-1* (5)	*SHV-187*	
ST218-3LV	*-*	*-*	*SHV-93*	
ST219 (2)	*CTX-M-15* (2)	*-*	*SHV-1*	
ST231 (2)	*CTX-M-15* (1)	*OXA-232* (1)	*SHV-1*	*ybt14* (1)*; iuc* (1)
ST237 (2)	*-*	*-*	*SHV-11*	
ST24-1LV	*-*	*-*	*SHV-11*	
ST2459 (1)	*CTX-M-15*	*-*	*SHV-1*	
ST2674 (1)	*CTX-M-15*	*NDM-1*	*SHV-11*	
ST2735 (1)	*-*	*-*	*SHV-11*	
ST29-1LV (1)	*CTX-M-15*	*-*	*-*	
ST292 (1)	*CTX-M-15*	*-*	*SHV-11*	
ST307 (6)	*CTX-M-15* (6)	*NDM-1* (4)	*SHV-28*	*ybt10* (3)
ST3161 (1)	*-*	*-*	*SHV-11*	
ST3430 (1)	*CTX-M-15*	*-*	*SHV-77*	
ST38 (1)	*CTX-M-15*	*NDM-1*	*SHV-11*	
ST383 (5)	*CTX-M-14* (4)*;CTX-M-15* (4)	*NDM-5* (4); *OXA-48* (4)	*SHV-1*	*iuc1* (4); *rmp1* (2)
ST39 (1)	*-*	*-*	*SHV-1*	*ybt4*
ST437 (7)	*CTX-M-15* (7)	*NDM-1* (1)*; NDM-5* (6)	*SHV-11*	*ybt9* (7)
ST45 (1)	*CTX-M-15*	*OmpK36* variant	*SHV-1*	*ybt10*
ST469 (1)	*CTX-M-15*	*-*	*SHV-11*	
ST474 (1)	*CTX-M-15*	*-*	*SHV-11*	
ST501 (1)	*CTX-M-15*	*-*	*SHV-11*	
ST514 (1)	*CTX-M-15*	*-*	*SHV-63*	
ST530 (2)	*CTX-M-15* (2)	*NDM-1* (2)	*-*	*ybt10* (2)
ST664 (2)	*CTM-X-15* (2)	*NDM-1* (2)	*SHV-11*	
ST882 (2)	*-*	-	-	*ybt* (2)
ST901 (1)	*CTX-M-15*	-	*SHV-1*	

ST: sequence type; ESBL: extended-spectrum β lactamase; Carb: carbapanemase; OmpK: outer-membrane porin substitutions contributing to carbapenem resistance; ybt: Yersiniabectin; Numbers in brackets indicate the number of isolates. Table S1 contains details of each isolate.

## Data Availability

The genome assemblies presented in this study are openly available in NCBI GenBank under BioProject ID PRJNA912084.

## Supplementary Materials

The following supporting information can be downloaded at: https://www.mdpi.com/article/10.3390/antibiotics12020233/s1. Figure S1: Minimum spanning tree (MST) of 84 *K. pneumoniae* by hospital information. Ridom SeqSphere+ MST for 84 samples based on 2358 columns, pairwise ignoring missing values, logarithmic scale. Cluster distance threshold: 15. Isolates grouped by colour indicate the different hospitals. Samples were collected from five different hospitals, and 37 different STs were identified, in addition to 14 transmission clusters, represented by shaded nodes and arrows. Numbers between the nodes indicate the number of allelic differences; Table S1: Sequence types, antimicrobial susceptibility and associated resistome; Table S2: Details of ST with virulence genes, K and O loci.
