# Supplementary material for: Klebsiella pneumonia in Sudan: Multidrug Resistance, Polyclonal Dissemination, and Virulence
Source: Antibiotics (Basel). 2023 Jan 21;12(2):233. doi: 10.3390/antibiotics12020233 (PMC9952582; doi:10.3390/antibiotics12020233)
Supplement: Supplementary file 1 [file antibiotics-12-00233-s001.zip › Figure S1.pdf]

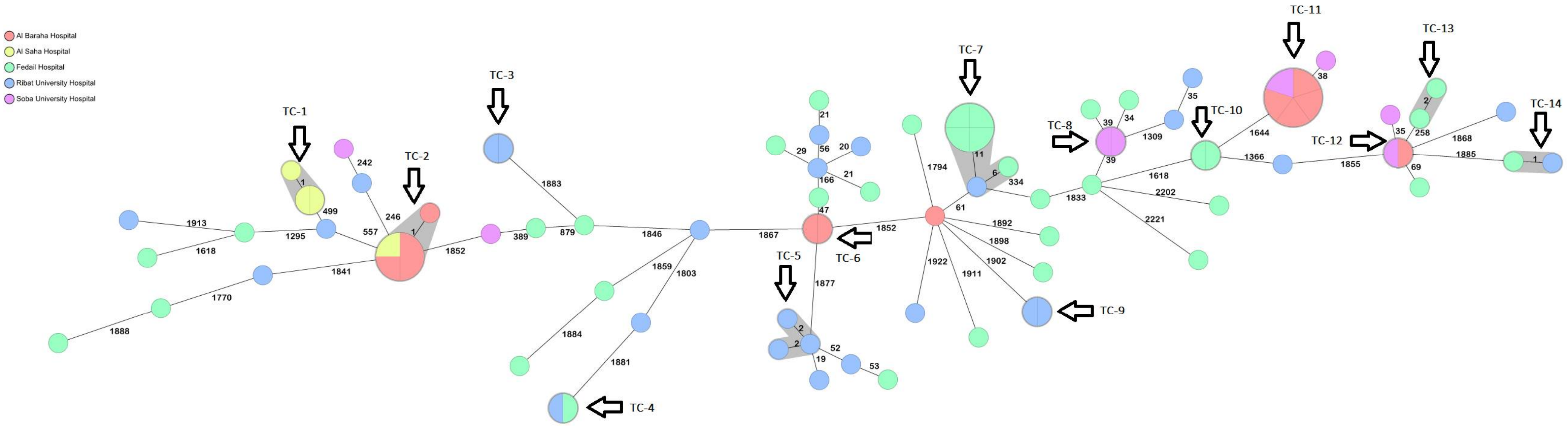

Figure S1: Ridom SeqSphere+ minimum spanning tree (MST) for 84 samples based on 2358 columns, pairwise ignoring missing values, logarithmic scale, *K. pneumoniae* MLST Pasteur (7). Cluster distance threshold: 15. Isolates grouped by colour indicating the different hospitals . Samples were collected from five different hospitals, 37 different STs were identified, in addition to 14 transmission clusters, represented by shaded nodes and arrows. Numbers between the nodes indicate the number of allelic differences.
