# Supplementary material for: Klebsiella pneumonia in Sudan: Multidrug Resistance, Polyclonal Dissemination, and Virulence
Source: Antibiotics (Basel). 2023 Jan 21;12(2):233. doi: 10.3390/antibiotics12020233 (PMC9952582; doi:10.3390/antibiotics12020233)
Supplement: Supplementary file 1 [file antibiotics-12-00233-s001.zip › Supplementary Table S1.pdf]

Table S1. Sequence types, antimicrobial susceptibility and associated resistome

ST	Sample name	AST phenotype	AGM R	Flq R	MLS R	PheR	Sul R	Tet R	Tmt R	bla R	ESBL	bla Carb R	Intrinsic β-lactamase	OmpK carbapenem R
**ST101**	HishK5	MDR, CR	*aac(3)-IIa; aadA; strA;strB*	*qnrS1*	-	*catII*	*sul2*	-	*dfrA14*	*OXA-1; TEM-1D*	*CTX-M-15*	*NDM-1*	*SHV-1*	**-**
	HishK7	MDR-CR	*aac(3)-IIa; strA;strB*	-	-	*catII*	*sul2*	*tet(D)*	*dfrA14*	*TEM-1D*	*CTX-M-15*	-	*SHV-1*	ompK35 substitution (G947A). Premature stop codon at amino acid position 316
	K11	MDR	*aac(3)-IIa; strA;strB*	-	-	*catII*	*sul2*	*tet(D)*	*dfrA14*	*TEM-1D*	*CTX-M-15*	-	*SHV-1*	
	K12	MDR	*aac(3)-IIa; strA;strB*	-	-	*catII*	*sul2*	*tet(D)*	*dfrA14*	*TEM-1D*	*CTX-M-15*	-	*SHV-1*	
	K5	MDR	*aac(3)-IIa; strA;strB*	-	-	*catII*	*sul2*	*tet(D)*	*dfrA14*	*TEM-1D*	*CTX-M-15*	-	*SHV-1*	
	K9	MDR	*aac(3)-IIa; strA;strB*	-	-	*catII*	*sul2*	*tet(D)*	*dfrA14*	*TEM-1D*	*CTX-M-15*	-	*SHV-1*	
**ST11**	LH_F139	MDR, CR	*aac(6')-Ib; aadA2; aph3-Ia;rmtF*	*qnrB1*	*mphA*	-	*sul1*	-	*dfrA12*	-	*CTX-M-15*	*NDM-4*	*SHV-11*	**-**
**ST1198**	LH_F137	S	-	-	-	-	-	-	-	-	-	-	*SHV-11*	**-**
**ST13**	LH_R290	MDR	*aac(3)-IIa*	-	-	-	-	-	-	*SCO-1; TEM-1D*	*CTX-M-15*	-	*SHV-1*	**-**
**ST1447**	LH_F68	S	-	-	-	-	-	-	-	-	-	-	*SHV-27*	**-**
**ST147**	K13	MDR	*aac(3)-IIa*	-	-	-	-	-	-	*SCO-1*	*CTX-M-15*	-	*SHV-11*	

	K3	MDR	*aac(3)-IIa*	-	-	-	-	-	-	*SCO-1*	*CTX-M-15*	-	*SHV-11*	
	LH_R289	MDR, CR	*aac(6')-Ib-cr; aph(3')-VI*	*qnrS1*	-	*catB3*	*sul1*	-	-	*OXA-1*	*CTX-M-15*	*NDM-1*	*SHV-11*	
	LH_R344	MDR, CR	*aac(6')-Ib'; aadA; aph(3')-VI; armA*	*qnrS1*	*mphA; mphE; msrE*	-	*sul1;sul2*	-	*dfrA5*	*OXA-9; TEM-1D*	*CTX-M-15*	*NDM-5*	*SHV-11*	
	LH_F18	MDR, CR	*aac(6')-Ib-cr; aph(3')-VI*	*qnrS1*	-	*catB3*	*sul1*	-	-	*OXA-1*	*CTX-M-15*	*NDM-1*	*SHV-11*	
	LH_F97	MDR	*aac(6')-Ib'; aad*	-	-	-	-	-	-	*OXA-9; TEM-1D*	-	-	*SHV-11*	
	LH_F134	MDR, CR	*aadA2; aph(3')-VI; strA;strB*	-	*mphA*	*catII*	*sul1*	-	*dfrA12*	-	*CTX-M-15*	*NDM-1*	*SHV-11*	
	LH_R387	MDR, CR	*aadA; aph(3')-VI*	*qnrS1*	*mphA*	*catB3*	*sul1*	-	-	*OXA-1; OXA-9; TEM-1D*	*CTX-M-15*	*NDM-1*	*SHV-11*	
	LH_F149	MDR, CR	*aadA; aph(3')-VI*	*qnrS1*	*mphA*	*catB3*	*sul1*	-	-	*OXA-1; OXA-9; TEM-1D*	*CTX-M-15*	*NDM-1*	*SHV-11*	
**ST15**	HishK3	MDR	*aac(3)-IIa; aac(6')-Ib-cr*	-	-	*catB4; catA1*	*sul1*	-	-	*CMY-4; OXA-1*	*CTX-M-15*	-	*SHV-28*	**-**
	HishK4	MDR	-	-	-	-	-	-	-	*CMY-4*	-	-	*SHV-28*	**-**
	K8	MDR	*aac(3)-IIa; aac(6')-Ib-cr*	-	-	*catA1*	*sul1*	-	-	*CMY-4*	*CTX-M-15*	-	*SHV-28*	**-**
	LH_F104	MDR	*aadA1*	-	-	-	*sul1*	*tet(A)*	*dfrA1*	*TEM-1D*	*CTX-M-15*	-	*SHV-28*	**-**
	LH_F122	MDR	*aadA1*	-	-	-	*sul1*	*tet(A)*	*dfrA1*	*TEM-1D*	*CTX-M-15*	-	*SHV-28*	**-**
	LH_F164	MDR, CR	*aac(3)-IIa; aac(6')-Ib-cr;*	*qnrS1*	*mphA*	*catB4; catA1*	*sul2*	*tet(A); tet(D)*	*dfrA14*	*OXA-1*	*CTX-M-15*	-	*SHV-28*	OmpK35 substitution (G690A).

			aph(3')-VI; strA;strB											Premature stop codon at amino acid position 230
**ST17**	HishK11	MDR	-	-	-	*catA1*	-	*tet(D)*	-	*TEM-1D*	-	-	*SHV-11*	**-**
	HishK12	MDR, CR	*aac(3)-IIa; aac(6')-Ib-cr; aadA5*	*qnrS1*	*mphA*	*catB4; catA1*	*sul1*	*tet(D)*	*dfrA17*	*OXA-1*	*CTX-M-15*	*NDM-1*	*SHV-11*	**-**
	HishK13	MDR	-	-	-	*catA1*	-	*tet(D)*	-	*TEM-1D*	-	-	*SHV-11*	**-**
	LH_R223	MDR	*strA;strB*	-	-	-	*sul2*	-	-	*TEM-1D*	*CTX-M-15*	-	*SHV-11*	**-**
**ST20**	HishK10	MDR	-	*qnrS1*	-	-	-	-	*dfrA14*	-	*CTX-M-15*	-	*SHV-187*	**-**
	K10	MDR, CR	*aac(3)-IIa; aac(6')-Ib-cr; aadA5; aph(3')-VI*	*qnrS1*	*mphA*	*catB0*	*sul1*	-	*dfrA17*	*OXA-1*	*CTX-M-15*	*NDM-1*	*SHV-187*	**-**
	HishK14	MDR, CR	*aac(3)-IIa; aac(6')-Ib-cr; aadA5; aph(3')-VI*	*qnrS1*	*mphA*	*catB1*	*sul1*	-	*dfrA17*	*OXA-1*	*CTX-M-15*	*NDM-1*	*SHV-187*	**-**
	K15	MDR, CR	*aac(3)-IIa; aac(6')-Ib-cr; aadA5*	*qnrS1*	*mphA*	*catB2*	*sul1*	-	*dfrA17*	-	*CTX-M-15*	*NDM-1*	*SHV-187*	**-**
	K2	MDR, CR	*aac(3)-IIa; aac(6')-Ib-cr; aadA5; aph(3')-VI*	*qnrS1*	*mphA*	*catB3*	*sul1*	-	*dfrA17*	*OXA-1*	*CTX-M-15*	*NDM-1*	*SHV-187*	**-**
	K6	MDR, CR	*aac(3)-IIa; aac(6')-Ib-*	*qnrS1*	*mphA*	*catB4*	*sul1*	-	*dfrA17*	*OXA-1*	*CTX-M-15*	*NDM-1*	*SHV-187*	**-**

			cr; aadA5; aph(3′)-VI											
	LH_R164	MDR	-	*qnrS1*	-	-	-	*tet(D)*	-	-	*CTX-M-15*	-	*SHV-187*	**-**
**ST218-3LV**	LH_R154	S	-	-	-	-	-	-	-	-	-	-	*SHV-93*	**-**
**ST219**	LH_F64	MDR	*strA;strB*	*qnrB4; qnrS1*	*mphA*	-	*sul1;sul2*	*tet(A)*	*dfrA14;dfrA17*	*DHA-1; TEM-1D*	*CTX-M-15*	-	*SHV-1*	**-**
	LH_F66	MDR	*strA;strB*	*qnrB4; qnrS1*	*mphA*	-	*sul1;sul2*	*tet(A)*	*dfrA14;dfrA17*	*DHA-1; TEM-1D*	*CTX-M-15*	-	*SHV-1*	**-**
**ST231**	LH_R92	MDR, CR	*aac(6′)-Ib; aadA2; rmtF*	*qnrS1*	*ermB; mphA*	-	*sul1*	-	*dfrA12*	*TEM-1D*	*CTX-M-15*	*OXA-232*	*SHV-1*	
	LH_R219	MDR	-	*qnrS1*	-	*catA1*	-	-	-	*TEM-1D*	-	-	*SHV-1*	
**ST237**	LH_R208	S	-	-	-	-	-	-	-	-	-	-	*SHV-11*	**-**
	LH_F281	S	-	-	-	-	-	-	-	-	-	-	*SHV-11*	**-**
**ST24-1LV**	LH_F2	S	-	-	-	-	-	-	-	-	-	-	*SHV-11*	**-**
**ST2459**	LH_F25	β-lactam resistant only	-	-	-	-	-	-	-	-	*CTX-M-15*	-	*SHV-1*	**-**
**ST2674**	LH_S25	MDR, CR	*aac(3)-IIa; aac(6′)-Ib-cr; aadA; aph(3′)-VI*	*qnrS1*	-	*catB4*	*sul1*	-	*dfrA15*	-	*CTX-M-15*	*NDM-1*	*SHV-11*	**-**
**ST2735**	LH_F50_1	MDR	*strA;strB*	*qnrB4*	-	-	*sul1*	-	-	*DHA-1*	-	-	*SHV-11*	**-**
**ST29-1LV**	LH_R314	MDR	-	-	-	*catA1*	-	-	*dfrA15*	*TEM-1D*	*CTX-M-15*	-	-	**-**
**ST292**	LH_F82	MDR	*strA;strB*	-	-	*catII*	*sul2*	-	*dfrA14*	*TEM-1D*	*CTX-M-15*	-	*SHV-11*	**-**
**ST307**	LH_R146	MDR, CR	*aac(3)-IIa; aadA;*	*qnrS1*	-	*catB1*	*sul2*	-	*dfrA14*	*OXA-1; TEM-1*	*CTX-M-15*	*NDM-1*	*SHV-28*	

			aph(3′)-VI; strA;strB											
	LH_R167	MDR, CR	*aac(3)-IIa; aph(3′)-VI; strA;strB*	*qnrS1*	-	*catB2*	*sul1;sul2*	-	*dfrA14;dfrA15*	*OXA-1; TEM-1*	*CTX-M-15*	*NDM-1*	*SHV-28*	
	LH_R182	MDR, CR	*aac(6′)-Ib-cr; aadA5; aph(3′)-VI; strA;strB*	*qnrB1; qnrS1*	*mphA*	*catB3*	*sul1;sul2*	*tet(A)*	*dfrA14;dfrA17*	*OXA-1*	*CTX-M-15*	*NDM-1*	*SHV-28*	
	LH_R174	MDR, CR	*aac(3)-IIa; aadA; aph(3′)-VI; strA;strB*	*qnrS1*	-	*catB4*	*sul2*	-	*dfrA14*	*OXA-1; TEM-1*	*CTX-M-15*	*NDM-1*	*SHV-28*	
	LH_R275	MDR	-	-	-	-	-	-	*dfrA14*	-	*CTX-M-15*	-	*SHV-28*	
	LH_F175	MDR	*aac(3)-IIa; aac(6′)-Ib-cr; strA;strB*	-	-	*catB4*	*sul2*	-	*dfrA14*	*OXA-1; TEM-1D*	*CTX-M-15*	-	*SHV-28*	
**ST3161**	LH_F86	S	-	-	-	-	-	-	-	-	-	-	*SHV-11*	**-**
**ST3430**	LH_R195	MDR	*aadA2; aph3-Ia; strA;strB*	*qnrS1*	*mphA*	-	*sul1;sul2*	*tet(A)*	*dfrA12*	-	*CTX-M-15*	-	*SHV-77*	**-**
**ST38**	LH_F158	MDR, CR	*aadA; aadA2; aph(3′)-VI; strA;strB*	*qnrS1*	*mphA*	*catB4*	*sul1;sul2*	-	*dfrA12;dfrA14*	*OXA-1*	*CTX-M-15*	*NDM-1*	*SHV-11*	
**ST383**	HishK8	MDR, CR	*aac(6′)-Ib′; aadA; aph3-Ia; armA; strA*	*qnrS1*	*mphA; mphE; msrE*	-	*sul1;sul2*	*tet(A)*	*dfrA5*	*TEM-1*	*CTX-M-14;CTX-M-15*	*NDM-5; OXA-48*	*SHV-1*	

	HishK9	MDR, CR	*aac(6′)-Ib′; aadA; aph3-Ia; strA*	*qnrS1*	*mphA; mphE*	-	*sul1;sul2*	*tet(A)*	*dfrA5*	*TEM-1*	*CTX-M-14;CTX-M-15*	*NDM-5; OXA-48*	*SHV-1*	
	LH_F15	MDR, CR	*aph3-Ia; strA;strB*	-	*mphA*	*catA1*	*sul2*	*tet(A)*	-	-	*CTX-M-14*	*OXA-48*	*SHV-1*	
	LH_F35	MDR, CR	*aac(6′)-Ib′; aadA; aph(3′)-VI*	*qnrS1*	-	-	-	-	-	*TEM-1*	*CTX-M-15*	*NDM-5*	*SHV-1*	
	LH_F190	MDR, CR	*aph(3′)-VI; aph3-Ia; armA; strA;strB*	*qnrS1*	*mphA; mphE; msrE*	-	*sul1;sul2*	*tet(A)*	*dfrA5*	-	*CTX-M-14;CTX-M-15*	*NDM-5; OXA-48*	*SHV-1*	**-**
**ST39**	LH_F146	S	-	-	-	-	-	-	-	-	-	-	*SHV-1*	**-**
	K4	MDR, CR	*aac(3)-IId; aac(6′)-Ib′; aadA; aph(3′)-VI*	*qnrS1*	-	-	-	-	-	*TEM-1*	*CTX-M-15*	*NDM-1*	*SHV-11*	**-**
	LH_R323	MDR, CR	*rmtB*	-	*ermB; mphA*	-	*sul1*	-	*dfrA30*	*TEM-1D*	*CTX-M-15*	*NDM-5*	*SHV-11*	
**ST437**	LH_F101	MDR, CR	*rmtB*	-	*ermB; mphA*	-	*sul1*	-	*dfrA30*	*TEM-1D*	*CTX-M-15*	*NDM-5*	*SHV-11*	
	LH_F102	MDR, CR	*rmtB*	-	*ermB; mphA*	-	*sul1*	-	*dfrA30*	*TEM-1D*	*CTX-M-15*	*NDM-5*	*SHV-11*	
	LH_F143	MDR, CR	*rmtB*	-	*mphA*	-	*sul1*	-	*dfrA30*	*TEM-1D*	*CTX-M-15*	*NDM-5*	*SHV-11*	
	LH_F169	MDR, CR	*rmtB*	-	*ermB; mphA*	-	*sul1*	-	*dfrA30*	*TEM-1D*	*CTX-M-15*	*NDM-5*	*SHV-11*	
	LH_F176	MDR, CR	*rmtB*	-	*ermB; mphA*	-	*sul1*	-	*dfrA30*	*TEM-1D*	*CTX-M-15*	*NDM-5*	*SHV-11*	

**ST45**	LH_R313	MDR-CR	*strA;strB*	*qnrS1*	-	-	*sul2*	-	*dfrA14*	*TEM-190*	*CTX-M-15*	-	*SHV-1*	ompK36 variant. LSP insertion at position 184
**ST469**	LH_F126	MDR	*aph3-Ia; strA;strB*	*qnrS1*	*mphA*	*catII*	*sul1; sul2*	*tet(A)*	*dfrA12*	-	*CTX-M-15*	-	*SHV-11*	**-**
**ST474**	LH_R384	MDR	*strA;strB*	*qnrB1*	-	-	*sul2*	-	*dfrA14*	*TEM-1D*	*CTX-M-15*	-	*SHV-11*	**-**
**ST501**	LH_F174	MDR	-	*qnrS1*	-	-	-	-	*dfrA14*	-	*CTX-M-15*	-	*SHV-11*	**-**
**ST514**	LH_R120	β-lactam resistant only	-	-	-	-	-	-	-	-	*CTX-M-15*	-	*SHV-63*	**-**
**ST530**	HishK1	MDR, CR	*aac(3)-IIa; aac(6′)-Ib-cr; aph(3′)-VI*	*qnrS1*	-	*catB4*	*sul2*	*tet(A); tet(D)*	*dfrA30*	*OXA-1*	*CTX-M-15*	*NDM-1*	-	**-**
	HishK2	MDR, CR	*aac(3)-IIa; aac(6′)-Ib-cr*	*qnrS1*	-	*catB4*	*sul2*	*tet(A); tet(D)*	*dfrA30*	*OXA-1*	*CTX-M-15*	*NDM-1*	-	**-**
**ST664**	LH_R100	MDR, CR	*aac(3)-IIa; aadA*	*qnrS1*	-	-	*sul1*	-	*dfrA15*	-	*CTX-M-15*	*NDM-1*	*SHV-11*	**-**
	LH_R107	MDR, CR	*aac(3)-IIa; aadA*	*qnrS1*	-	-	*sul1*	-	*dfrA15*	-	*CTX-M-15*	*NDM-1*	*SHV-11*	**-**
**ST882**	LH_R162	S	-	-	-	-	-	-	-	-	-	-	*SHV-101*	**-**
	LH_F192	S	-	-	-	-	-	-	-	-	-	-	*SHV-101*	**-**
**ST901**	LH_F159	MDR	*aac(6′)-Ib-cr; aadA; strA;strB*	*qnrS1*	-	*catB4*	*sul1*	-	*dfrA15*	*OXA-1; TEM-1D*	*CTX-M-15*	-	*SHV-1*	**-**

ST: sequence type; AST: antimicrobial susceptibility test; AGly: aminoglycoside; R: resistance; Flq: Fluoroquinolone; MLS: Macrolide; Phe: Chloramphenicol; Sul: sulphonamide; Tet: tetracycline; Tmt: trimethoprim; Bla: β-lactamase; ESBL: extended-spectrum β lactamase; Carb: carbapanemase; OmpK: outer-membrane porin substitutions contributing to carbapenem resistance; MDR: multidrug resistance; CR: carbapenem resistant; S: Susceptible to all tested antibiotics.

OmpK35 and ompK36 sequences were analysed using Geneious Prime. OmpK35 Accession no KX528047.1 was used as a reference for OmpK35 sequence analysis. Reference laboratory strain ATCC43816 (Accession no CP009208.1), and amino acid sequences 6CRCP and 6CRD3 on Protein Data Bank (PDB) **10.2210/pdb6rcp/pdb** [15] were used as references for OmpK36 sequence analysis.
