# Supplementary material for: Klebsiella pneumonia in Sudan: Multidrug Resistance, Polyclonal Dissemination, and Virulence
Source: Antibiotics (Basel). 2023 Jan 21;12(2):233. doi: 10.3390/antibiotics12020233 (PMC9952582; doi:10.3390/antibiotics12020233)
Supplement: Supplementary file 1 [file antibiotics-12-00233-s001.zip › Supplementary Table S2.pdf]

Table S2: Details of ST with virulence genes, K and O loci

ST	Sample name	Yersiniabactin	Aerobactin	RmpADC	K_locus	O_locus
ST101	HishK5	ybt 9; ICEKp3	-	-	unknown (best match = KL107)	O1v1
	HishK7	ybt 9; ICEKp3	-	-	unknown (best match = KL107)	O1v1
	K11	ybt 9; ICEKp3	-	-	unknown (best match = KL17)	O1v1
	K12	ybt 9; ICEKp3	-	-	unknown (best match = KL17)	O1v1
	K5	ybt 9; ICEKp3	-	-	KL17	O1v1
	K9	ybt 9; ICEKp3	-	-	unknown (best match = KL107)	O1/O2v1
ST11	LH_F139	ybt 9; ICEKp3	-	-	KL15	O4
ST1198	LH_F137	-	-	-	KL3	O2v2
ST13	LH_R290	-	-	-	KL3	O1v2
ST1447	LH_F68	-	-	-	KL115	O3b
ST147	K13	-	-	-	unknown (best match = KL10)	O3/O3a
	K3	-	-	-	KL10	O3/O3a
	LH_R289	ybt 9; ICEKp3	-	-	KL64	O2v1
	LH_R344	ybt 9; ICEKp3	-	-	KL64	O2v1
	LH_F18	ybt 9; ICEKp3	-	-	KL64	O2v1
	LH_F97	ybt 9; ICEKp3	-	-	KL64	O2v1
	LH_F134	-	-	-	KL10	O3/O3a
	LH_R387	ybt 9; ICEKp3	-	-	unknown (best match = KL107)	OL101
	LH_F149	ybt 9; ICEKp3	-	-	unknown (best match = KL107)	OL101

ST15	HishK3	-	-	-	unknown (best match = KL48)	O1v1
	HishK4	-	-	-	unknown (best match = KL50)	unknown (best match = O1/O2v1)
	K8	-	-	-	unknown (best match = KL107)	O1v1
	LH_F104	-	-	-	KL112	O1v2
	LH_F122	-	-	-	KL112	O1v2
	LH_F164	ybt 9; ICEKp3	-	-	KL24	O1v1
ST17	HishK11	-	-	-	unknown (best match = KL107)	unknown (best match = O2v1)
	HishK12	-	-	-	unknown (best match = KL122)	O2v2
	HishK13	-	-	-	unknown (best match = KL122)	O2v2
	LH_R223	-	-	-	KL23	O2v2
ST20	HishK10	-	-	-	unknown (best match = KL28)	O1v2
	K10	-	-	-	unknown (best match = KL64)	O1v1
	HishK14	-	-	-	unknown (best match = KL64)	O1v1
	K15	-	-	-	unknown (best match = KL64)	O1/O2v1
	K2	-	-	-	KL64	O1v1
	K6	-	-	-	unknown (best match = KL64)	O1v1

	LH_R164	-	-	-	KL28	O1v2
ST218-3LV	LH_R154	-	-	-	KL112	O2v2
ST219	LH_F64	-	-	-	KL114	O1v1
	LH_F66	-	-	-	KL114	O1v1
ST231	LH_R92	ybt 14; ICEKp5	iuc unknown	-	KL51	O1v2
	LH_R219	-	-	-	KL51	O1v2
ST237	LH_R208	-	-	-	KL21	O2v1
	LH_F281	-	-	-	KL21	O2v1
ST24-1LV	LH_F2	-	-	-	KL2	O1/O2v2
ST2459	LH_F25	-	-	-	KL60	O3b
ST2674	LH_S25	-	-	-	KL110	O2v1
ST2735	LH_F50_1	-	-	-	KL110	O1v1
ST29-1LV	LH_R314	-	-	-	KL63	O1v2
ST292	LH_F82	-	-	-	KL50	O3b
ST307	LH_R146	ybt 10; ICEKp4	-	-	KL102	O2v2
	LH_R167	ybt 10; ICEKp4	-	-	KL102	O2v2
	LH_R182	-	-	-	KL102	O2v2
	LH_R174	ybt 10; ICEKp4	-	-	KL102	O2v2
	LH_R275	-	-	-	KL102	O2v2
	LH_F175	-	-	-	KL102	O2v2
ST3161	LH_F86	ybt 10; ICEKp4	-	-	KL55	O3/O3a
ST3430	LH_R195	-	-	-	KL52	OL101
ST38	LH_F158	-	-	-	KL52	OL101
ST383	HishK8	-	iuc 1	-	unknown (best match = KL30)	unknown (best match = O1v2)
	HishK9	-	iuc 1	-	unknown (best match = KL30)	O1/O2v1
	LH_F15	-	-	-	KL30	O1/O2v2
	LH_F35	-	iuc 1	rmp 1; KpVP-1	KL30	O1v2
	LH_F190	-	iuc 1	rmp 1; KpVP-1	unknown (best	O1v2

					match = KL30)	
ST39	LH_F146	ybt 4; plasmid	-	-	KL149	O2v2
ST437	K4	ybt 9; ICEKp3	-	-	KL36	O4
	LH_R323	ybt 9; ICEKp3	-	-	KL36	O4
	LH_F101	ybt 9; ICEKp3	-	-	KL36	O4
	LH_F102	ybt 9; ICEKp3	-	-	KL36	O4
	LH_F143	ybt 9; ICEKp3	-	-	KL36	O4
	LH_F169	ybt 9; ICEKp3	-	-	KL36	O4
	LH_F176	ybt 9; ICEKp3	-	-	KL36	O4
ST45	LH_R313	ybt 10; ICEKp4	-	-	KL62	O2v1
ST469	LH_F126	-	-	-	KL139	O3b
ST474	LH_R384	-	-	-	KL17	O4
ST501	LH_F174	-	-	-	KL10	O3b
ST514	LH_R120	-	-	-	KL35	O1v1
ST530	HishK1	ybt 10; ICEKp4	-	-	unknown (best match = KL54)	unknown (best match = O1v2)
	HishK2	ybt 10; ICEKp4	-	-	unknown (best match = KL113)	unknown (best match = O1/O2v1)
ST664	LH_R100	-	-	-	KL62	O1v1
	LH_R107	-	-	-	KL62	O1v1
ST882	LH_R162	ybt 4; plasmid	-	-	KL16	O1v1
	LH_F192	ybt 4; plasmid	-	-	KL16	O1v1
ST901	LH_F159	-	-	-	KL3	O2v2
